# Evaluating large language models for AI-assisted grading: a framework and case study in higher education

**DOI:** 10.1038/s41598-026-48656-3

**Published:** 2026-04-18

**Authors:** Yago Saez, Luis Mario Garcia, Asuncion Mochon, Pedro Isasi

**Affiliations:** 1 Computer Science Department, Universidad Carlos III de Madrid, Avda. de la Universidad 30, Madrid, 28911 Madrid Spain; 2 Department of Applied Economics and Economic History, UNED, Paseo Senda del Rey, Madrid, 28040 Madrid Spain

**Keywords:** Large Language Models (LLMs), AI-assisted grading, Higher education, Evaluation framework, Educational assessment, Feedback quality, Benchmarking Methodology, Instructional technology, Engineering, Mathematics and computing

## Abstract

This article presents an empirical evaluation of six state-of-the-art large language models for grading student assignments in a university-level course on data analytics and machine learning. The study compares the ability of the models to generate grades and feedback with that of human instructors, using statistical and semantic measures for evaluation. The results show that DeepSeek-R1 provided the closest alignment with human evaluations in both grading accuracy and feedback quality. Beyond this case study, the article contributes a replicable framework for systematically benchmarking LLMs in higher education assessment, specifying model selection, prompt design, evaluation measures, and cost analysis. The proposed framework ensures continued relevance as new models emerge, providing educators and researchers with a transferable methodology to evaluate AI-assisted grading in higher education.

## Introduction

This research aims to provide higher education professors with a comprehensive reference for selecting the most suitable large language model (LLM) to support their assessments in data analytics and machine learning (ML). Through the analysis of several LLMs we are comparing advantages, limitations, challenges, and potential issues associated with different LLMs, this study seeks to facilitate informed decision-making in academic evaluation processes.

The remainder of this paper is organized as follows. Section State of the art reviews related work on AI-assisted assessment and identifies current challenges in the field. Section Methodology presents the proposed evaluation framework, describes the case study context, and details the methodology used in this study. Section Analysis and discussion of results reports the results of applying the framework to six LLMs and discusses the findings in relation to existing literature, highlights limitations, and reflects on pedagogical and institutional implications. Finally, Section Conclusions concludes the paper and outlines directions for future research.

### Motivation

Assessment remains one of the most resource-intensive elements of teaching, particularly in courses with large cohorts such as data analytics and machine learning. Instructors face the dual challenge of providing timely, individualized feedback while maintaining consistency and fairness. Technology-enhanced approaches, including LLMs, offer a potential pathway to scale assessment processes, but their reliability and pedagogical value remain in question.

Recent studies have highlighted both the potential and limitations of AI-assisted grading^1^. Manual assessment, though usually accurate, is resource intensive and can be biased. Automated Grading Tools (AGTs) offer efficiency, but often lack the ability to provide qualitative information or award partial marks. However, AI-assisted grading has demonstrated the ability to bridge this gap, offering timely and unbiased evaluations. The recent advent of LLMs, along with the continuous advancements, is redefining the landscape of educational assessment. These technologies enhance the system’s ability to process complex language constructs, adapt to varied instructional designs, and maintain a consistent evaluative framework, thus marking a significant step forward in the automation of pedagogical tasks, such as assessing code correctness and quality, comparing student’s performance with predefined rubrics, and generating constructive feedback. Tools such as *TA Buddy*, ChatGPT, *GRAD-AI*, and *Gipy* have shown promise in grading programming tasks, detecting errors, and ensuring fairness in evaluations. Despite these advances, challenges remain, including potential biases, hallucinated feedback, and the need for human oversight (see^2–5^).

To explore how these limitations affect the assessment process and to offer an updated overview of latest technologies, this research explores the performance of state-of-the-art LLM versions for automated evaluation of data analytics and machine learning assignments. By testing multiple LLM models, we aim to assess their effectiveness in providing accurate, consistent, and unbiased grading, as well as meaningful feedback. The integration of AI-driven assessment tools has the potential to significantly enhance the grading process, offering instant feedback, and reducing the workload on educators. Moreover, as society increasingly embraces AI-based solutions, the adoption of automated grading tools will be essential to promote a more interactive and adaptive learning environment.

## State of the art

### The integration of AI in education

In recent years, AI has become increasingly relevant in education, transforming teaching, learning, and assessment processes. Recent studies highlight its growing role in intelligent tutoring systems, adaptive learning environments, and AI-driven assessment tools. The review^6^ analyzes the use of AI for student assessment in a total of 454 papers included in Scopus and Web of Science. The authors emphasize how AI supports formative assessment and automated grading, allowing personalized feedback and reducing the workload of instructors. Similarly, the study^7 ^categorizes AI applications into development, application, and integration layers, showcasing how AI-powered feedback, reasoning, and adaptive learning enhance educational experiences. These authors selected a total of 100 articles from the education and educational research category of the Social Sciences Citation Index database from 2010 to 2020. In a broader perspective^8,9^, analyze two decades of AI research in education, revealing trends in intelligent tutoring, educational data mining, and recommendation systems for personalized learning.

The study^10^ reviews the trends, adoption measures, diverse applications and current limitations of ChatGPT research in higher education, including research articles published between 2023 and 2024. Research conducted by^11^ further explores AI’s role in teaching, focusing on the relationship between the supply of professional development and the demand for AI integration among teachers. A notable advancement is the rise of conversational AI, particularly chatbots and virtual assistants, which facilitate real-time student’s interaction. This work^12^ discusses how these systems improve engagement by providing natural, human-like communication.

Despite these advances and opportunities, significant challenges remain^13^. The authors highlight concerns regarding “pedagogical value”, noting that although LLMs can approximate human grading in technical tasks, they often lack the nuanced understanding of a student’s individual learning trajectory. Additional concerns also persist in relation to ethical considerations, data privacy, and instructor preparedness, underscoring the need for continued research and sustained professional development to ensure a responsible integration of AI in education. Nevertheless, AI’s potential to enhance personalization and efficiency in educational processes continues to grow, positioning it as a major driver of innovation in the field.

#### AI-Powered feedback and automated grading in education

In the field of education, feedback is defined as information provided by an agent, such instructor or peer, regarding to a learner’s performance or understanding, and it represents one of the most relevant factors influencing student learning^14^. In other related work, synthesis defines formative feedback as information intended to modify learners’ thinking or behaviour, and remarks that effective formative feedback should be timely and specific^15^.

Recent advances in AI applied to generate feedback and automated assessments has increasingly gained attention in education. AI-driven tools, particularly those that leverage generative models, offer scalable solutions for grading, feedback delivery, and evaluation of student’s performance. These tools have the potential to automatically generate individualized feedback at a low cost for educators^13^.

This study^16^ explores students’ perceptions of AI-generated feedback, showing that students struggle to differentiate between AI and human feedback in some cases. However, once the source is disclosed, students tend to prefer human feedback and rate AI-generated feedback lower in credibility and genuineness. The work^17^ examines AI’s role in automating grading, highlighting the shift from traditional grading methods to AI-driven assessment. Their study underscores AI’s ability to improve feedback efficiency while raising concerns about fairness and ethical considerations.

The research^18^ introduces *Smart Grading*, an AI-based system that automates text-based answer evaluation using LLMs. This tool allows educators to specify grading rubrics, sample solutions, and evaluation criteria, demonstrating high reliability in expert validation studies. Similarly, there is another study that explores how master’s students in translation engage with ChatGPT-generated feedback during their revision process^19^. To further investigate this area, the work^20^ assess the effectiveness of GPT-3.5 and GPT-4 in generating feedback from the evaluation. Their findings indicate that AI-generated feedback is more structured and consistent than human feedback, with GPT-4 performing better in terms of reliability and comprehensiveness.

Although AI-generated feedback has been studied in terms of linguistic quality and student perceptions, there is not a clear consensus on how to evaluate its pedagogical value in a reproducible way. This gap reinforces the importance of a framework that combines lexical, semantic, and contextual measures to benchmark feedback quality across models.

### AI for computer science and programming

The integration of AI into computer science education has significantly evolved, progressing from early automated assessment systems to AI-powered tutoring and feedback mechanisms. Research has increasingly explored AI’s role in facilitating programming instruction, personalizing learning experiences, and reducing instructor workload.

Early studies emphasized AI-driven evaluation and feedback mechanisms. The study^21^ explores the use of LLMs to generate programming exercises and explanations, highlighting their ability to support students’ learning while alleviating instructor workload, although there remains a need for oversight to ensure the quality of the generated content. Another related work compared the performance of university students and GPT-3.5 and GPT-4 in physics coding assignments^22^. Following this work,^23^discusses the dual impact of generative AI on computing education, highlighting challenges such as students’ overreliance and academic integrity alongside opportunities for personalized learning and innovative teaching. It stresses the need for educators to adapt curricula to integrate these tools effectively and responsibly.

Rapid advancements in AI-generated code have gained attention, and it is one of the main concerns in programming education. The work^24^ developed ML models to detect AI-generated pseudocode in high school programming courses, identifying key differences between human and AI-generated code. The rise of AI-generated code tempts a shift in the first course of Computer Science (CS) major towards high-level abstraction, but^25^ advocates teaching early CS learners to understand AI tools, not just use them to avoid creating insecure “black box” code. Furthermore, an investigation found that students’ interaction with ChatGPT during programming tasks varied by guidance level, with prompt training significantly improving AI use and learning outcomes^26^. Aligned with previous, the study^27^ showed that ChatGPT had limited impact on beginners’ programming performance, while intermediate students perceived greater benefits from its use. Shifting to the professional perspective, the survey^28 ^gathered insights from 410 developers on AI programming assistants, finding that they help efficiency, but often fail to meet functional needs. Key challenges include usability issues and difficulty controlling outputs, suggesting improvements for better adoption. In a completely different use case^29^, evaluated the use of AI systems to automatically determine the time complexity of the student’s code. Finally^30^, conducted a review of the literature based on the use of ChatGPT’s application in programming education, identifying benefits such as personalized tutoring, real-time feedback, and knowledge reinforcement while also highlighting risks of over-reliance and academic dishonesty.

#### AI-Assisted grading and feedback in programming

Before the adoption of AI-based grading systems, numerous automated assessment tools were developed to address the challenges of evaluating programming assignments at scale. Early systems such as *AutoGrader*^31^, and *Drop Project*^32^, focused on correctness and efficiency using static and dynamic analysis. Reviews like^33–35^ highlighted the limitations of these tools, such as usability issues, limited support for higher-order code features, and a predominant focus on functional correctness. More recent developments, including *ProGrader*^36^, and *DebugProGrade*^37^, have incorporated feedback generation, test-driven development, and debugging assessment. Despite these advances, most tools lacked the flexibility and contextual understanding that AI systems are nowadays beginning to address.

As in other fields, the use of AI for automating the evaluation of programming assignments has also gained traction with LLMs, significantly reducing instructor workload while providing structured and scalable assessment solutions. AI-driven grading tools leverage ML models plus LLMs to evaluate source code, provide feedback, and assess broader learning objectives such as coding style, logic correctness, and even collaboration skills. For example^38^, explored GPT-3’s feedback on Python assignments, finding high variability in accuracy, indicating that AI cannot yet fully replace human grading, and^39^ applied ML to assess collaboration in programming projects, showing that AI can support grading but requires human oversight. The work^2^ found that GPT-3.5 hints improved student’s performance but led to over-reliance on AI feedback, and^3^ introduced *GRAD-AI*, an ML-based grading tool that enhances accuracy and feedback timeliness. The study^40^ evaluated ChatGPT’s grading of Python assignments, showing a strong correlation with human grading but raising concerns about inconsistencies. Another related work compared LLM-generated feedback with automated test-based feedback, finding AI useful but prone to inaccuracies^41^.

The work^42^found that students in a Java programming course preferred instructor feedback over AI-generated feedback, as it led to greater score improvements, highlighting the need for hybrid feedback models. Similarly^4,5^, introduced *TA Buddy*, an AI-assisted grading tool that reduced the grading time by 24% while maintaining 90% agreement with manual grading, demonstrating AI’s potential in large-scale programming courses.

Despite these advances, challenges remain in fully automating programming evaluation. AI-generated assessments must be carefully designed to align with learning objectives, minimize bias, and ensure that students develop essential problem-solving skills rather than over-relying on AI assistance. This motivates the need for a systematic framework that standardizes model selection, prompt design, and evaluation measures.

Future research should focus on refining hybrid models that integrate AI automation with human oversight, which can be properly tested with a standard framework, ensuring accuracy, fairness, and pedagogical alignment in programming education.

## Methodology

This section outlines the methodological approach adopted in the study. It presents a first version of the proposed framework for evaluating LLMs in higher education assessment, designed to ensure replicability and transferability across contexts. Then it describes the specific case study in which the framework was applied, including course setting, student assignments, selected models, and prompt design. Finally, it details the evaluation procedures and metrics used to compare AI-generated and human-generated grades and feedback.

### Proposed framework for evaluating LLMs in higher education assessment

Given the rapid evolution of LLMs, a comparison of individual models is necessarily short-lived. In contrast, a structured framework can remain relevant over time, guiding future evaluations as new models emerge and as institutions consider adoption.

The framework of this study builds on previous research in automated assessment and educational technology, and it explicitly integrates three perspectives:

1. **Technical performance of the models:** The proposed framework generates both numerical grades and textual feedback. Grading performance is evaluated by comparing model-generated scores with instructor-assigned grades. We report descriptive statistics and conduct an independent-samples *t*-test to examine mean differences. In line with prior work on LLM-based automated grading^43,44^, we further compute Mean Absolute Error (MAE) to quantify score deviation. The quality of generated feedback is assessed using established Natural Language Generation (NLG) evaluation metrics, comparing model-generated feedback with the instructor-provided feedback, following standard NLG evaluation practices^45^.
2. **The pedagogical value of AI-generated outputs:** According to^13^, assessment through LLMs in education shall account for feedback quality, and impact for students and instructors. It also has limitations that require for continuous human oversight. In this context, with open-ended questions and code, pedagogical value of feedback is not easy to evaluate, and it transcends mere textual similarity. The study^14 ^proposes a model of feedback (feed up/back/forward) and levels (task, process, self-regulation and self), where the pedagogical “power of feedback” depends on whether it supports learning goals, next steps, the discussion of the timing of feedback, and the effects of positive and negative feedback. With a similar perspective^46^, connect feedback to self-regulated learning and outline seven principles of good feedback practice in higher education, supporting the pedagogical value dimension beyond numeric marks agreement. In addition to numeric metrics such as ROUGE scores and BERTScore^47^, proposes a five-dimension framework that accounts for readability, suggestions, problems, positive tone and factuality, for manually evaluating system-generated feedback. Similarly^48^, manually evaluated the quality of the AI-generated compared to human’s feedback, across five pedagogically relevant dimensions: criteria-based alignment, clarity of directions, accuracy of content, coverage of essential features, and supportive tone. To take into consideration these multidimensional perspectives, our framework adopts a two-fold evaluation strategy: 1) a quantitative assessment of lexical and semantic similarity metrics relative to expert benchmarks, and 2) a post-hoc qualitative instructor review. This dual-perspective method is designed to cross-validate automated performance against expert pedagogical judgment, specifically accounting for the “verbosity expansion” common in generative models and the nuances of open-ended tasks. By combining numeric metrics with expert oversight, the framework ensures a “human-in-the-loop” verification process, acknowledging that while AI can produce valuable and similar feedbacks, human expertise is essential for validating contextual depth, and ensuring an appropriate and constructive tone for student’s feedback.
3. **Institutional considerations**: Our framework accounts for institutional constraints such as cost, transparency, and data protection compliance. The proposed procedures are aligned with our institutional internal policies but also take into account international AI governance guidelines, generative AI safeguards, and human-centered oversight in education^49^, NIST Trustworthy and Responsible AI Report^50^. To accomplish with these standards, this study was formally approved by the Chair of the institutional office of undergraduate studies and academic quality committee, and informed consent was obtained from all participants. All student artifacts were fully anonymized prior to processing and stored in secure, encrypted environments in accordance with GDPR guidelines. Additionally, we used enterprise-grade LLM APIs that explicitly prohibit the use of input data for model training. To ensure pedagogical safety, all model outputs underwent manual review to identify and mitigate any instances of unethical content, biased language, or inappropriate instructional tones.

Table 1 summarizes the proposed framework and illustrates how each element was designed in the present study. The framework is structured around seven components: model selection criteria, prompt design and parameters, evaluation measures for grades, evaluation measures for feedback, handling of variability, cost estimation, and reporting and interpretation. Each element is described in general terms, and an example is provided to demonstrate its application in this case study. Although this work presents a first version of the framework, and it is focused on assignments in data analytics and machine learning, the framework is intentionally designed to be transferable across disciplines and institutional contexts.

**Table 1:** Proposed framework for evaluating LLMs in higher education assessment.

Framework element	Description	Example in this study
Model selection criteria	Define rationale for choosing models (e.g., performance, accessibility, licensing, institutional relevance).	Six models selected: GPT-4o, GPT-4o-mini, Llama-3.1 and 3.3 (70B), DeepSeek-V3, DeepSeek-R1. Mix of proprietary and open-source models.
Prompt design & Parameters	Specify how grading instructions are formulated; document style, iterations, and parameters.	Single structured prompt designed iteratively. Temperature fixed at 0 for deterministic outputs. Appendix B contains full text.
Evaluation measures – Grades	Identify quantitative measures for comparing model-generated vs. human grades.	Mean Absolute Error (MAE), Pearson/Spearman correlation, independent-samples *t*-tests, and Wilcoxon signed-rank tests.
Evaluation measures – Feedback	Define a two-fold strategy: 1) Multi-level similarity metrics (Lexical, Embedding, ROUGE, BERTScore) and 2) Post-hoc qualitative analysis	Quantitative: Lexical (Cosine/Jaccard), Embeddings (Word2Vec/spaCy), ROUGE-1/2/L (Recall-focused), and BERTScore (rescaled). Qualitative: Instructor’s review of depth and tone.
Handling variability & Verbosity	Describe treatment of non-determinism and length differences (e.g., verbosity expansion).	Temperature control; evaluation of “verbosity expansion” (AI vs. Manual length); Recall-oriented metrics to account for length disparity.
Institutional Compliance	Ensure alignment with data protection (GDPR), ethical guidelines, and institutional policies.	Ethics committee approval; full anonymization; use of enterprise APIs (no-training clause); manual audit for bias/tone.
Cost & Practical Reporting	Estimate practical adoption costs and bridge technical outcomes with pedagogical implications.	API pricing vs. GPU costs. Results translated into “Human-in-the-loop” workflows and instructor workload impact.

### Case study context

For this research we have used 110 students’ deliverables from the course Big Data and Business Analytics from the University Carlos III of Madrid. The selected assignments are related to data analytics and machine learning topics. This course was selected due to its large student enrollment and the open-ended Python notebook assignments, which demand significant time and effort for thorough evaluation.

In this specific course, students submit deliverables in the form of Python notebooks, which consist of data analysis and machine learning exercises (Practice 1 and 2), which address open questions. The objective of these deliverables is to evaluate how students apply their knowledge, leaving them freedom to explore different approaches as long as they are appropriate and properly argued. Professors provide detailed written feedback and assign a score out of 100, a process that, while valuable, is time-consuming and not scalable. To explore the potential of generative AI in this context, this research investigates the use of LLMs to assist in the evaluation of these deliverables.

### Evaluation procedure

The evaluation procedure follows a structured methodology to ensure that both manual and automated evaluations are conducted systematically. Initially, all student deliverables are collected and manually evaluated by professors. Subsequently, the Python notebooks pass through an anonymization process, where all personal identifiers are removed, and file names are encrypted to maintain confidentiality. Once the anonymization process is completed, each deliverable is submitted to the selected LLMs using a predefined evaluation prompt that will be the same for all students, but slightly different per type of practice. The LLMs generate two outputs: a written review and a numerical grade, both of which are systematically stored for further analysis. After all submissions have been processed, a comparative analysis is performed using a script that calculates different metrics and similarity scores from the LLM-generated feedback and grades compared to the manual evaluations made by the professor.

**Fig. 1:**
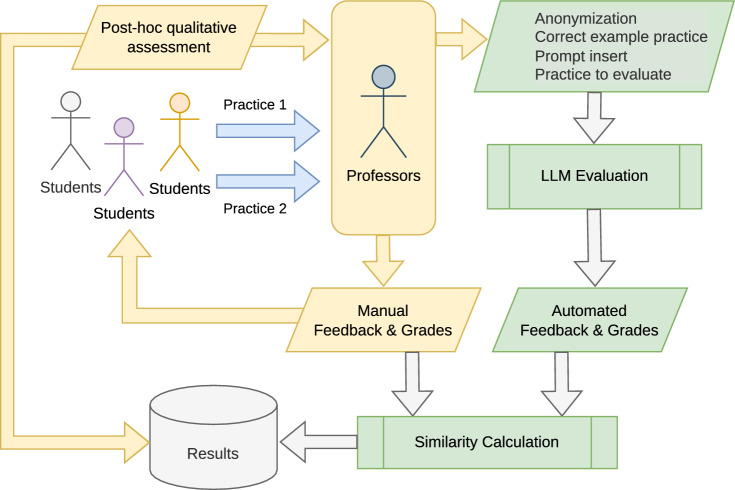
Schema of the methodology implemented.

See Fig. 1 for an illustration of the methodology conducted for this study.

#### Input

During the course, students complete two different deliverables. Practice 1 (P1) focuses on data analytics, requiring students to handle multiple datasets, perform Exploratory Data Analysis (EDA), and answer open-ended questions based on their findings. Practice 2 (P2) involves applying ML concepts, where students must develop and evaluate different models to analyze past data and determine the best strategies for designing, training, testing and evaluating adequate ML models.

To ensure data confidentiality and compliance with the GDPR^1^ regulations, an anonymization process has been developed to prepare the input for LLMs. This process involved removing from the notebooks any personal information, such as names, emails and students’ IDs. To maintain traceability while ensuring anonymity, we generated an encrypted hash for each file name. This hashed identifier was then used for request processing and result storage, effectively dissociating any identifiable student’s information from the practices.

Once the data was anonymized, we asked the instructors to give their assessment guidelines, and starting from these evaluation guides, we explored several prompt formulations to optimize the performance of the model. After some basic empirical experimentation, we ended up selecting the prompts included in Appendix B.

#### LLM selection

As mentioned earlier in this study, we focus on evaluating student’s deliverables in the form of Python notebooks, which contain complex, structured responses that require in-depth analysis of code, markdown explanations, and data outputs. Given the length of these deliverables (averaging 50,139.51 (±38,877) tokens^2^ per submission) a context window of at least 128 K tokens is recommended to process each submission atomically, without requiring chunking strategies that could disrupt coherence and evaluation accuracy^3^. This implies that, rather than partitioning the data into smaller subsets and managing their retrieval individually, the model can process the prompt and the entire notebook to perform the evaluation across each complete deliverable at once.

The selected generative AI models represent a balanced mix of state-of-the-art proprietary models and cost-effective or open-source alternatives that can be deployed locally. This selection ensures a fair comparison between cutting-edge AI capabilities and more accessible solutions.

1. **GPT-4o & GPT-4o-mini (OpenAI)**: These models represent the current highest performing proprietary LLMs, with multimodal capabilities (GPT-4o^4^) and cost-efficient alternatives (GPT-4o-mini^5^). They are special editions of GPT-4, designed to be even better at certain tasks. The “o” in GPT-4o stands for optimization. They are known for having high accuracy, strong reasoning, and coherence; however, these models are not open source, with a limited control over fine-tuning and potential API costs.
2. **Llama 3.1-70B**^6^ & **Llama 3.3-70B**^7^** (Meta AI)**: These models provide open-source alternatives that allow for local deployment, improving transparency and control. They are cost-effective (when self-hosted) and highly customizable; however, running them locally at quantization FP8 or FP16 requires high-end hardware (GPUs with large VRAM, like A100 or H100). These models are quantized to FP8, which makes them efficient, but may result in slightly lower precision in text generation compared to full-precision models (FP16).
3. **DeepSeek-R1**^8^ & **DeepSeek-V3**^9^**(DeepSeek)**: These models offer strong coding performance and retrieval-augmented capabilities, which may enhance evaluation of Python notebooks. They are open source models, so they allow the research community to refine better models in the future. DeepSeek-R1, incorporates cold-start data before the Reinforcing Learning (RL) process, and DeepSeek-V3 uses a Mixture-of-Experts (MoE) approach. They have proven to be competitive with GPT-4o across math, code, and reasoning tasks, showing in addition, potential cost savings. Since they are quite new, they are less tested in educational contexts and present a potential variance in performance across different programming tasks. Both have been used quantized to FP8.

**Table 2:** Selected Generative AI models.

Model	Developer	Parameters	Quantization	Context Length	Cost*
GPT-4o	OpenAI	N/A	N/A	128K	$2.50/$10
GPT-4o-mini	OpenAI	N/A	N/A	128K	$0.15/$0.60
Llama 3.1-70B	Meta AI	70 Billion	FP8	131K	Free - $0.18/$0.18
Llama 3.3-70B	Meta AI	70 Billion	FP8	128K	Free - $0.88/$0.88
DeepSeek-R1	DeepSeek	685 Billion	FP8	164K	Free - $3/$7
DeepSeek-V3	DeepSeek	671 Billion	FP8	131K	Free - $1.25/$1.25

*Current estimated cost per million tokens (input/output) based on publicly available API pricing or usage tiers. “Free” indicates availability as open-source software. Local deployment needs to comply with latest minimum hardware requirements.

Selected generative AI models are described in Table 2. As can be seen some of them are quantized to FP8, enhancing their speed, We assume that GPT-4o runs at full precision FP16, although specific details have not been publicly disclosed.

#### Alternative models

While current model selection prioritizes a balance between state-of-the-art performance, affordability, and deployability, additional models were considered. The Mistral 7B/8x22B, Gemma and Qwen models were excluded due to their limited token context windows (32K), which does not meet the study’s requirements. On the other hand, Google’s Gemini 1.5 Pro, with its 1M token context, Grok-1.5, xAI’s latest model, and Anthropic’s Claude 3 Opus, remain potential candidates for future research and evaluation.

### Evaluation

To rigorously determine the viability of LLMs as automated grading assistants, a multi-dimensional evaluation framework is employed. This assessment transcends simple score comparison by analyzing the models through three primary dimensions: statistical reliability of numerical grades, the linguistic and pedagogical quality of textual feedback, and the economic feasibility of deployment. By systematically comparing the AI-generated outputs against human-validated benchmarks for both practices (P1 and P2), we aim to identify not only which models most closely mirror human judgment but also which offer the most sustainable balance between instructional depth and computational cost. This holistic approach ensures that the “best” model is selected based on a synthesis of accuracy, semantic alignment, and practical scalability.

Once the different LLMs have been used to assess both deliverables, an objective analysis of the results is necessary. For each student and each practice (P1 and P2), we obtain three key outputs: the final grade, the textual feedback provided by the model, and the number of input/output tokens consumed and produced by the models. These outputs are systematically compared to evaluate the consistency, accuracy, relevance of the assessments and costs across all different models.

#### Numeric grade value

We have performed a statistical evaluation of the numeric grade values assigned during the evaluation process. This analysis allows us to quantify the consistency and accuracy of the grading model in comparison to reference scores provided by human evaluators. More specifically, we have analyzed most common measures of central tendency, mean, median and mode; dispersion and variability metrics: standard deviation, range and interquartile range. We also calculated the average absolute difference between LLMs and manual grades, providing a direct measure of the grading error, that is, the Mean Absolute Error (MAE). The most relevant metrics are summarized in Table 3. Finally, a comparison of the AI-generated values with the manual grades by using statistical significance tests has been conducted, results are summarized in Table 4.

#### Textual assessment quality

The quality of textual feedback is examined through both quantitative metrics and a complementary manual post-hoc qualitative analysis, enabling a balanced evaluation of semantic alignment and pedagogical characteristics.

***a) Textual assessment metrics***

In addition to numeric evaluation, to assess the quality and effectiveness of the written feedback generated by the model, several evaluation metrics based on the taxonomy established by^45^ have been selected. These different metrics are calculated with the aim of capturing different aspects of the similarity and relevance of the written outputs, the hybrid evaluation approach incorporates both the lexical and semantic similarity measures.

1. **Lexical similarity metrics:** this computes the basic fundamental distance-based measures to quantify the overlap between the generated feedback and the manual reference text^51^. These metrics provide a straightforward assessment of textual similarity based on token matching:
  Cosine similarity: This metric calculates the cosine of the angle between two text vectors in a high-dimensional space, reflecting their similarity based on word frequency distributions. Higher values indicate greater similarity^52^.
  Jaccard similarity: Defined as the ratio of the intersection to the union of word sets, this measure evaluates the degree of word overlap between the generated feedback and the manual text^53^.
2. **Embedding-Based similarity measures:** To capture semantic similarity beyond exact word matching, word and sentence embedding techniques using Natural Language Toolkit (NLTK) and pre-trained models have been used:
  Word embedding similarity (*Word emb*): Each word in the text is mapped to a continuous vector space using a pre-trained word embedding model^10^. The similarity is then computed as the average of the cosine similarities between word vectors in the manual and AI-generated feedback.
  Sentence embedding similarity (*Sentence emb*): Instead of individual words, sentence-level embeddings^11^ provide a holistic representation of the text. As before, the similarity scores are calculated as the average of the cosine similarities between the embedded representations of the manual and AI-generated feedback sentences.
3. **ROUGE score analysis:** To evaluate the quality of feedback in terms of content coverage, we applied the Recall-Oriented Understudy for Gisting Evaluation (ROUGE) metrics, commonly used in text summarization and machine-generated text comparisons^55^:
  *ROUGE1*: Measures the overlap of uni-grams (single words) between the generated feedback and reference text, providing a basic lexical match assessment.
  *ROUGE2*: Measures the overlap of bi-grams (two consecutive words) between the generated feedback and reference text, providing an assessment of short phrase-level lexical similarity.
  *ROUGEL*: Captures the longest common subsequence (LCS), which accounts for in-sequence word matches while allowing for gaps, thus reflecting more flexible textual similarity.
  ROUGE metrics evaluates similarity using precision, recall, and F1. Considering the substantial difference in output size, where manual feedback is shorter than AI-generated feedback, we focus exclusively on recall, since it best reflects whether essential reference content is preserved in the generated text.
4. **BERT Score**: This learned metric was originally developed to measure how similar a text summary is to the original text^56^. This score measures the similarity between tokens in two text sequences by representing them as BERT embeddings and computes the similarity of two sentences as a sum of cosine similarities between their tokens’ embeddings. While n-gram-based metrics, such as ROUGE, rely on exact token matches and overlapping word sequences, BERTScore leverages BERT contextual embeddings from transformer-based models^57^. This approach allows this metric to capture semantic relationships and contextual meaning for deeper text similarities, making it more effective when language variations and in a context where nuanced interpretation of text is essential^58^. For the interpretation of BERTScore it’s also important to remark that the extensive word-length disparity between the manual feedback and the LLM-generated outputs influences the semantic similarity metrics. Since BERTScore uses pre-normalized vectors, then computed scores have the numerical range of cosine similarity (between −1 and 1). Although this does not affect BERTScore’s ability to rank text generation systems, it makes the absolute score values less interpretable. In practice, the observed scores fall within a relatively narrow range, possibly due to the learned geometry of contextual embeddings. To deal with this inconvenient, the authors of the BERTScore, in order to increase the score readability, suggests rescaling. Rescaling adjusts this metric with an in-domain random-pair as baseline, providing a practical lower-bound reference for semantic alignment in this dataset. As before, this technique assess similarity through precision, recall, and F-measure values, shown as *BERTScore P*, *BERTScore R* and *BERTScore F1*. For the BERTScore calculation, we used *distilbert-base-uncased-distilled-squad* as the underlying transformer model. While standard BERT models provide a general linguistic baseline, the SQuAD^12^ finetuned variant is specifically optimized for extractive semantic tasks, making it superior at identifying shared pedagogical intent between concise human references and verbose AI-generated feedback, its distilled architecture prioritizes core semantic signals over linguistic noise.

Including all these diverse evaluation techniques into our analysis allows to capture both surface-level and semantic correspondences, providing a robust framework for evaluating the effectiveness of the outcome generated by the model compared to the manual feedback.

Finally, an Aggregate Score (*z* mean) has been calculated, as the mean of z-scores across all metrics for each model. These evaluation results are collected in Tables 5 and 6.

***b) Post-hoc qualitative analysis***

To better understand the results and potential sources of variation in grading outcomes, we conducted a post-hoc qualitative review of the dataset. While such analyses often rely on sampled subsets due to dataset size, our dataset comprised 660 scorable comparisons with both manual and automated grades and feedback available (110 deliverables per model tested), allowing an initial screening of all cases. A first instructor performed a preliminary review to identify patterns of agreement, divergence, and potential methodological issues. Based on this screening, a stratified sampling strategy was defined for the qualitative analysis.

From the full dataset (660 scorable comparisons), 243 cases (36.81%) were selected for detailed qualitative examination using a stratified sampling strategy designed to emphasize edge cases while preserving a representative baseline. The adjudication set included: (i) all cases with large grade divergence, defined as absolute grade differences above the third quartile (Q3) of the observed distribution (156 cases, 23.63%); (ii) a simple random sample corresponding to approximately 15% of the remaining cases to provide a representative baseline of typical grading agreement (71 cases, 10.75%); and (iii) methodological divergence cases defined as instances with highly similar grades (absolute difference $<5$ points) but low semantic similarity in feedback (BERTScore F1 below the first quartile, Q1), resulting in 16 cases (2.4%). Counts reported here correspond to the aggregated dataset for all models across both practices^13^.

Each selected case was reviewed by two instructors who examined both grades and feedback to identify factors contributing to scoring differences. The instructors analyzed the cases independently. When disagreements occurred, they were resolved through discussion until consensus was reached. Initial agreement across categories was 81.48%. The analysis focused on informative but shallow feedback, minor but important mistakes missed, subtle methodological arguments not captured, and subjective divergences between instructor and model evaluations. The findings reported are derived from this qualitative analysis of 243 cases and should therefore be interpreted as descriptive and hypothesis-generating rather than confirmatory. In Section "Post-hoc qualitative analysis", where relevant, we report the number of cases supporting specific observations and provide references to illustrative examples.

#### Cost analysis

The cost evaluation was performed by estimating the total number of input and output tokens required per practice and applying each model provider’s published pricing rates per one million tokens, which distinguish between input and output usage. For open-source models, pricing was calculated using their publicly reported API rates to maintain consistency in the comparison framework, while acknowledging that local deployment may alter operational costs depending on available hardware infrastructure. This procedure enables a uniform and reproducible economic assessment across models. As described before in Table 2, pricing is based on real average token usage, with separate rates for input and output tokens, and open-source models can be deployed locally if hardware requirements are met. The average cost per practice based on token usage are reported in Table 8.

## Analysis and discussion of results

This section presents a brief analysis of the results obtained from the evaluation of the selected LLMs in three dimensions: numerical grading accuracy, feedback quality, and computational cost.

### Numerical grading results

The statistical analysis of the numerical grades highlights that only DeepSeek-R1 consistently aligns with manual grading across both practices. Specifically, DeepSeek-R1 shows no statistically significant differences (p > 0.01) when compared to human evaluations, as confirmed by both paired *t*-tests and Wilcoxon signed-rank tests. As can be seen in Table 3, DeepSeek-R1 MAE remained close to 19 points in both assignments, outperforming all other models. In contrast, other models such as GPT-4o and the Llama series exhibited consistent higher grades and larger deviations, especially in P1, indicating a less reliable evaluation in open-ended data analytics tasks. Overall, the models consistently assigned higher grades than the instructors, indicating a systematic tendency to overgrade.

Since Shapiro-Wilk Test for normality does not reject normality with confidence (p-value P1: 0.1279 and p-value P2: 0.1835) we have performed parametric *t*-test paired tests to find if the differences found are statistically significant. Both statistical significance approaches obtained very similar results. In the case of P1, only the DeepSeek-R1 model shows no statistical significance difference compared to manual grades. For P2, all models except Llama-3.1 and Llama-3.3, achieve similar results, showing not statistical significance differences from manual grades. T-statistic shows closest value to 0 for DeepSeek-R1 in both P1 and P2, see Table 4 for more details. To complete the analysis, and for having a more robust outcome, we also performed non-parametric Wilcoxon Signed-Rank tests, finding exactly the same conclusions as with the paired *t*-test tests, results are in Appendix Table 9.

**Table 3:** Summary of mean (with % deviation), standard deviation, MAE for P1 and P2 grades respect to manual grade.

Practice	Model	Mean	% diff respect Manual	Std Dev	MAE
P1	gpt4oMini	71.46	+31.0%	6.13	23.18
P1	gpt4o	72.14	+32.3%	5.95	23.17
P1	Llama-3.1	74.43	+36.5%	9.69	24.18
P1	Llama-3.3	75.58	+38.6%	9.54	24.11
P1	DeepSeek-R1	**57.47**	+5.4%	10.33	**19.17**
P1	DeepSeek-V3	66.99	+22.8%	7.44	20.15
P1	Manual Grade	54.53		23.81	-
P2	gpt4oMini	73.71	+9.8%	4.06	20.44
P2	gpt4o	72.50	+7.9%	6.09	**18.79**
P2	Llama-3.1	88.64	+32.0%	8.43	24.69
P2	Llama-3.3	89.93	+33.9%	7.12	24.34
P2	DeepSeek-R1	**68.50**	+2.0%	8.24	19.01
P2	DeepSeek-V3	72.54	+8.0%	7.81	19.47
P2	Manual Grade	67.16		23.40	-

**Table 4:** Paired *t*-test comparing LLMs grades to manual grades.

Practice	Model	t-statistic	p-value	Significant (99%)
P1	gpt4oMini	−6.0355	0.0000	Yes
P1	gpt4o	−6.6203	0.0000	Yes
P1	Llama-3.1	−7.3357	0.0000	Yes
P1	Llama-3.3	−8.2970	0.0000	Yes
P1	DeepSeek-R1	**−1.0696**	**0.2884**	**No**
P1	DeepSeek-V3	−4.9995	0.0000	Yes
P2	gpt4oMini	−1.4053	**0.1713**	**No**
P2	gpt4o	−1.2133	**0.2355**	**No**
P2	Llama-3.1	−4.9933	0.0000	Yes
P2	Llama-3.3	−5.2587	0.0000	Yes
P2	DeepSeek-R1	**−0.3058**	**0.7621**	**No**
P2	DeepSeek-V3	−1.2662	**0.2162**	**No**

### Textual evaluation quality

#### Textual assessment metrics

When assessing feedback quality by quantitative metrics, DeepSeek-R1 also stood out by achieving the highest Aggregated Scores (*z*-score), combining lexical, semantic, and BERT-based similarity metrics. Its feedback was closer to the human benchmark not only in structure and coherence, but also in capturing the main insights expected by educators. However, as observed in the post-hoc quantitative analysis, even the best-performing models struggled sometimes when identifying minor but pedagogically relevant mistakes. These oversights explain the observed MAE values and underline a key limitation: LLMs tend to underplay subtle errors that educators would typically penalize, especially in tasks requiring interpretative reasoning or methodological justification.

The experimental results collected in Tables 5 and 6 indicate that one of the proposed models, DeepSeek-R1, consistently achieves the highest textual metrics when compared to manual feedback in both practices, positioning it as the most suitable LLM for AI-assisted grading in our study case.

**Table 5:** Average and standard deviation of automated feedback compared to manual for P1.

	gpt4o	gpt4oMini	Llama-3.1	Llama-3.3	DeepSeek-R1	DeepSeek-V3
Metric						
Cosine	0.364 (±0.067)	0.363 (±0.072)	0.330 (±0.070)	0.346 (±0.084)	**0.372** (±0.063)	0.356 (±0.076)
Jaccard	0.056 (±0.019)	0.056 (±0.019)	0.061 (±0.022)	0.062 (±0.021)	**0.066** (±0.023)	0.061 (±0.021)
Word emb	0.646 (±0.030)	0.657 (±0.031)	0.625 (±0.029)	0.630 (±0.029)	**0.681** (±0.037)	0.645 (±0.031)
Sentence emb	0.954 (±0.015)	0.954 (±0.014)	0.954 (±0.015)	0.949 (±0.018)	**0.961** (±0.014)	0.942 (±0.020)
ROUGE1	0.166 (±0.056)	0.149 (±0.050)	0.342 (±0.093)	0.352 (±0.073)	**0.578** (±0.074)	0.401 (±0.084)
ROUGE2	0.014 (±0.009)	0.015 (±0.008)	0.039 (±0.032)	0.040 (±0.026)	**0.067** (±0.031)	0.041 (±0.030)
ROUGEL	0.078 (±0.020)	0.070 (±0.018)	0.204 (±0.071)	0.189 (±0.052)	**0.284** (±0.082)	0.206 (±0.064)
BERTScore P*	0.334 (±0.098)	0.320 (±0.104)	0.350 (±0.139)	0.369 (±0.053)	**0.392** (±0.061)	0.356 (±0.105)
BERTScore R*	**0.374** (±0.060)	0.366 (±0.062)	0.323 (±0.127)	0.342 (±0.081)	0.346 (±0.106)	0.305 (±0.118)
BERTScore F1*	0.354 (±0.078)	0.343 (±0.083)	0.336 (±0.130)	0.356 (±0.066)	**0.369** (±0.084)	0.331 (±0.109)
Aggregate Score (*z* mean)	−0.28	−0.41	−0.35	0.01	**1.32**	−0.30

*BERTScore: distilbert-base-uncased-distilled-squad_L4_no-idf_version=0.3.12-rescaled.

**Table 6:** Average and standard deviation of automated feedback compared to manual for P2.

	gpt4o	gpt4oMini	Llama-3.1	Llama-3.3	DeepSeek-R1	DeepSeek-V3
Metric						
Cosine	0.358 (±0.071)	**0.372** (±0.071)	0.302 (±0.133)	0.331 (±0.108)	0.324 (±0.072)	0.349 (±0.087)
Jaccard	0.051 (±0.019)	0.049 (±0.013)	0.045 (±0.022)	0.051 (±0.019)	**0.055** (±0.017)	0.053 (±0.021)
Word emb	0.661 (±0.027)	0.664 (±0.026)	0.602 (±0.126)	0.624 (±0.080)	**0.688** (±0.030)	0.647 (±0.027)
Sentence emb	**0.957** (±0.011)	0.957 (±0.013)	0.900 (±0.140)	0.934 (±0.096)	0.949 (±0.018)	0.935 (±0.028)
ROUGE1	0.142 (±0.038)	0.128 (±0.037)	0.333 (±0.140)	0.367 (±0.102)	**0.488** (±0.049)	0.372 (±0.070)
ROUGE2	0.011 (±0.006)	0.010 (±0.007)	0.025 (±0.018)	0.031 (±0.022)	**0.037** (±0.022)	0.034 (±0.022)
ROUGEL	0.067 (±0.013)	0.060 (±0.013)	0.169 (±0.073)	0.180 (±0.052)	**0.227** (±0.040)	0.193 (±0.051)
BERTScore P*	0.335 (±0.058)	0.329 (±0.049)	0.261 (±0.247)	0.329 (±0.141)	**0.380** (±0.040)	0.357 (±0.039)
BERTScore R*	**0.367** (±0.039)	0.361 (±0.038)	0.254 (±0.186)	0.310 (±0.122)	0.345 (±0.059)	0.312 (±0.059)
BERTScore F1*	0.351 (±0.048)	0.344 (±0.043)	0.258 (±0.217)	0.320 (±0.131)	**0.363** (±0.048)	0.335 (±0.047)
Aggregate Score (*z* mean)	0.03	−0.04	−1.12	−0.04	**0.85**	0.33

*BERTScore: distilbert-base-uncased-distilled-squad_L4_no-idf_version=0.3.12-rescaled.

#### Post-hoc qualitative analysis

The exploratory post-hoc qualitative review found no evidence of harmful bias, unethical language, or inappropriate or degrading tone in any of the assessments. Instead, the feedback was consistently constructive, well-structured, and positive.

As anticipated, the results showed that model-generated feedback was substantially more verbose than instructor comments. Across all deliverables, manual feedback averaged approximately 133–135 words (166–168 tokens), and all models exceeded this length by more than four times in average token count. DeepSeek-R1 generated particularly extensive responses, reaching nearly six times the length of instructor feedback. Overall, the findings indicate a systematic expansion in verbosity across all evaluated LLMs compared to manual feedback.

Nevertheless, the verbosity of the AI-generated feedback was generally informative, often providing valuable and clear explanations. In the instructor adjudication of the stratified subsample (243 cases), 194 cases (79.8%) were classified as informative but lacking contextual depth by at least one instructor. In these cases, the feedback covered multiple rubric dimensions and often included actionable suggestions, but it failed to capture the deeper reasoning highlighted in the instructor’s evaluation.

In the evaluation subset, instructors identified other recurring limitations: minor but important mistakes were missed in 148/243 cases (60.9%), subtle methodological arguments in 189/243 (77.8%), and subjective judgment divergences in 156/243 (64.2%). These issues typically occurred in situations where the LLM grade was substantially higher than the manual reference despite concrete defects in the submission or where the instructor feedback relied on reasoning about the analytical workflow that was not reflected in the automated evaluation. These findings are consistent with the nature of the evaluated practices, which included open-ended analytical questions requiring nuanced interpretation and therefore greater subjectivity than closed-ended tasks.

When analyzing performance per model across these feedback dimensions, DeepSeek-R1 outperformed the other models. Table 7 presents the results of the two-instructor evaluation on the annotated subsample, detailed by LLM^14^.

**Table 7:** Two-instructor evaluation results on the stratified subsample.

Model	N	Informative but shallow	Minor important	Subtle methodological	Subjective divergence
gpt4o	35	29/35 (82.9%)	22/35 (62.9%)	29/35 (82.9%)	23/35 (65.7%)
gpt4oMini	37	32/37 (86.5%)	25/37 (67.6%)	32/37 (86.5%)	26/37 (70.3%)
Llama-3.1	52	41/52 (78.8%)	34/52 (65.4%)	42/52 (80.8%)	34/52 (65.4%)
Llama-3.3	47	36/47 (76.6%)	33/47 (70.2%)	36/47 (76.6%)	33/47 (70.2%)
DeepSeek-R1	36	**26/36** **(72.2%)**	**11/36** **(30.6%)**	**21/36** **(58.3%)**	**17/36** **(47.2%)**
DeepSeek-V3	36	30/36 (83.3%)	23/36 (63.9%)	29/36 (80.6%)	23/36 (63.9%)

As shown in Table 7, the instructor’s evaluations reveal clear differences between the evaluated LLMs, with DeepSeek-R1 consistently outperforming the other models. To better illustrate the qualitative patterns summarized above, we present representative examples for each of the four recurring feedback limitations identified during the instructor adjudication.

***a) Informative but shallow feedback***

In many cases, the models produced feedback that appeared detailed and helpful, but failed to capture the reasoning underlying the instructor’s evaluation. One representative summary of this example, which originally contains 435 words, is:

**DeepSeek-V3, P1, case 61**:’...The code runs without errors and the data loading and merging steps are correctly implemented, but variable names are vague and the workflow lacks sufficient documentation. The analysis relies mainly on basic t-tests and visual comparisons and could benefit from more robust statistical methods such as ANOVA or regression. While visualizations are clear, some lack proper labels or context, and the treatment of missing values or outliers is not fully addressed. (...)’

The model-generated feedback was clearly informative in form, addressing all rubric dimensions (not fully reproduced in the example for brevity, but quite similar to the example included in the Appendix C), including code correctness, data handling, analysis, visualization, and documentation, and it offered concrete and pedagogically useful suggestions for improvement. On the surface, this makes the feedback appear complete and reasonably thorough. However, the manual review expressed a much deeper concern. Rather than focusing only on technical weaknesses, the human evaluator questioned whether the student actually understood the analytical process, noting unnecessary repeated file loading, improper data cleaning, not fully correct use of statistical testing, and conclusions partially based on invalid data. This case illustrates how LLM feedback may satisfy formal expectations of clarity and helpfulness while still missing the broader contextual judgment that the work was methodologically unsound and incomplete.

***b) Minor but important mistakes missed***

In other cases, the models overlooked concrete defects that were explicitly identified in the instructor review. For instance, in Practice 1 (P1) a large discrepancy was observed between the manual and DeepSeek-R1 grades ($+42$). The instructor’s manual evaluation identified several critical data-cleaning failures that rendered the student’s analysis invalid: the student failed to perform necessary date format transformations, did not load numeric data properly (overlooking decimal separator issues), and neglected to check and remove important data errors such as products missing prices or impossible profit margins (exceeding 1).

In contrast, the AI-generated feedback provided a more vague critique without specific actionable directions:

**DeepSeek-R1, P1, case 43**: ’...Data handling is inadequate. They merged datasets but didn’t standardize date formats properly. The 2024 data only goes up to August, but the student didn’t adjust the analysis period for fair year-over-year comparisons, which could skew results.(...)’

Although the model correctly identified the date standardization issue, it did not explain how the flawed transformation invalidated the downstream analysis. This illustrates a gap in functional specificity, where the model sometimes recognizes a problem but fails to diagnose the underlying technical failure and its consequences.

***c) Subtle methodological arguments not captured***

A frequent limitation involved reasoning about methodological validity rather than isolated technical issues. For example, in this case, the instructor explained that the comparison between two predictive models was methodologically invalid because the evaluation metrics were computed over differently grouped data, and the LLM provided several generic recommendations, but it did not recognize the underlying reasoning flaw that invalidated the comparison.

**Llama 3.1, P2, case 9**: ’...The models are combined correctly and the reasoning behind their selection is explained. However, the evaluation could be improved through better parameter tuning (e.g., grid search or cross-validation) and additional metrics such as confusion matrices or classification reports. Visualizations are generally clear but would benefit from improved labeling and additional techniques such as distributions or heatmaps. Further comments and documentation would also improve the clarity of the workflow.(...)’

As shown in the example, the model correctly identified several relevant issues but failed to capture the central argument of the manual review: the apparent superiority of the Decision Tree model proposed by the student was an artifact of data aggregation rather than a valid analytical result.

***d) Subjective divergence***

Finally, divergences were also observed in subjective aspects of the evaluation, such as organization, clarity, and overall understanding of the task. For example, in P1 case 33 (gpt4oMini), the instructor penalized the submission for poor organization, repeated overwriting of intermediate results, and lack of clarity in the analytical workflow. In contrast, the model produced a more positive evaluation, emphasizing that the notebook demonstrated a reasonable understanding of the task. Although the feedback appeared constructive, it did not reproduce the instructor’s critical assessment of the student’s analytical reasoning.

**gpt4oMini, P1, case 33**: ’...The code generally runs correctly and the data is handled appropriately, though some redundancy and variable naming could be improved. The analysis methods are valid but would benefit from stronger statistical support and clearer structure. Visualizations are relevant but inconsistent in color usage, and the notebook would gain from more detailed documentation explaining the analytical steps and results.(...)’

As shown in Table 7, many other assessments fall into one or more of these categories. Additional representative summarized examples for each LLM are provided in Appendix A.

### Cost-efficiency considerations

From a deployment perspective, the cost analysis reveals substantial differences between models. While GPT-4o offers strong performance, its high cost per million tokens ($2.50/$10 input/output) may limit scalability in real-world academic environments. Conversely, open-source alternatives like Llama 3.1 or DeepSeek-V3 are considerably more affordable, with DeepSeek-V3 representing a balanced trade-off between cost and performance. Although DeepSeek-R1 is expensive per API call, its open-source availability and superior feedback quality position it as a strong candidate for scalable, AI-assisted grading systems, provided the necessary computational resources are in place.

On average, these practices involve 50,139.51 input tokens and 443.96 output tokens. Accordingly, the average cost per practice is reported in Table 8.

**Table 8:** Average cost per practice based on token usage.

Model	Developer	Input/Output Cost (per 1M)	Open Source	Avg. Cost per Practice
gpt4o	OpenAI	$2.50/$10	No	$0.1316
gpt4oMini	OpenAI	$0.15/$0.60	No	**$0.0079**
Llama-3.1	Meta AI	$0.18/$0.18	Yes	$0.0092
Llama-3.3	Meta AI	$0.88/$0.88	Yes	$0.0450
DeepSeek-R1	DeepSeek	$3/$7	Yes	$0.1582
DeepSeek-V3	DeepSeek	$1.25/$1.25	Yes	$0.0652

Attending to these cost figures, the differences are substantial and may have notable implications when scaling assessment systems to a high volume of deliverables. However, the advantages, such as enhanced feedback quality, reduced evaluation time, improved support for instructors, and faster turnaround for student’s feedback, represent valuable gains that could justify the investment.

### Discussion

One of the key strengths observed in AI-generated assessments is their ability to provide immediate complete and comprehensive feedback. Traditional manual grading, although diligent and rigorous, often suffers from delays, which can impact students’ ability to iterate on their learning cycle. The quick evaluation offered by LLMs facilitates timely feedback, allowing students to refine their understanding and improve future submissions with continuous and incremental learning. Moreover, the models demonstrated a high level of consistency in grading, reducing the subjectivity or bias that can sometimes arise in human assessments.

However, our findings also reveal notable challenges associated with AI-enhanced grading. A primary concern is the variability in grading accuracy across different models. While models such as GPT-4o or DeepSeek-R1 exhibited strong alignment with human evaluations, other models, particularly Llama alternatives, showed higher deviations. This discrepancy underscores the importance of selecting robust LLMs and possibly fine-tuning them to align with pedagogical objectives. Statistical analysis of AI-generated grades indicates that while central tendency measures (mean, median) align closely with human evaluations, dispersion metrics (standard deviation, interquartile range) suggest a potential tendency of models to either over-penalize or over-reward certain aspects of assignments.

When evaluating the quality of textual feedback, lexical and embedding-based similarity measures showed that AI-generated comments were generally comprehensive but, as observed in the exploratory post-hoc analysis, some cases lacked specificity.

These findings suggest that future research of AI grading tools could benefit from domain-specific prompt engineering, model fine-tuning or reinforcement learning strategies to enhance feedback quality but still with post-hoc manual supervision.

Although the rescaled BERTScores demonstrate a semantic alignment significantly above the stochastic baseline, the numerical range is moderated by the ’Precision Penalty’ inherent in the metric’s greedy matching algorithm. This is because BERTScore calculates precision by averaging the similarity scores for every candidate token, the abundance of additional pedagogical detail in the LLM responses, which often provide more exhaustive justification than the concise human “gold standard” constrained by time, results in numerous tokens with no direct semantic counterpart in the reference.

However, it is important to acknowledge that semantic similarity does not inherently equate to pedagogical quality, for that we did post-hoc analysis. Extensive verbosity suggests the model AI-generated feedback may provide added valuable pedagogical support for students, but semantic similarity and verbosity does not necessarily equate to pedagogical effectiveness.

Importantly, this proposed framework is intended to evaluate AI-assisted assessment, where the LLM generates both grades and feedback that are used as drafts that instructors must review to ensure pedagogical standards.

In overall, the results support the viability of integrating advanced LLMs into the educational workflow, particularly for formative assessment tasks. Integration of LLMs not only improves efficiency but also promotes formative assessment practices, allowing for iterative students’ learning and real-time pedagogical adaptation core priorities in technology-enhanced learning environments. However, the observed variability in accuracy, cost, and semantic depth of feedback reinforces the need for selective deployment, rigorous model benchmarking, and, most importantly, a hybrid approach that leverages human oversight to address the nuanced aspects of student’s evaluation.

#### Educational and institutional interpretation

Although numerical analyses and similarity metrics provide rigorous evidence of model performance, their implications extend directly to pedagogical practice and institutional policy in higher education. Three main interpretations emerge:

1. **Pedagogical implications:** The findings show that DeepSeek-R1 consistently aligns with human evaluations and produces feedback with high semantic similarity. Pedagogically, this translates into more reliable formative assessment, where students receive timely, actionable feedback that closely mirrors instructor expectations. Faster turnaround supports iterative learning cycles, allowing students to review misconceptions before subsequent assignments. At the same time, the tendency of LLMs to overlook subtle but pedagogically relevant errors highlights the need for hybrid assessment workflows: AI can efficiently provide first-pass grading and structured feedback, while instructors retain oversight for nuanced judgments and can provide good information about student’s progress.
2. **Instructor workload and teaching practices:** For educators, the adoption of LLMs offers a tangible reduction in grading time, especially in large cohorts where traditional manual assessment is unsustainable. By automating repetitive elements of feedback, instructors can redirect effort toward higher-value pedagogical tasks such as mentoring, personalized guidance, and curriculum innovation. This shift aligns with current trends in technology-enhanced higher education that emphasize teaching presence over administrative grading labor.
3. **Institutional and policy considerations:** From an institutional perspective, the study highlights important trade-offs between performance and cost. While proprietary models such as GPT-4o provide strong results, their scalability is limited by API costs. Open-source alternatives such as DeepSeek-V3 offer a more cost-effective pathway to institutional adoption, particularly when deployed on university-managed infrastructure. These findings suggest that universities should consider hybrid adoption strategies, balancing performance, affordability, and ethical compliance. Furthermore, the adherence of the methodology to GDPR through anonymization demonstrates that AI-assisted grading can be implemented within existing regulatory frameworks, a critical requirement for institutional policy and quality assurance in higher education. As part of the framework procedure, the post-hoc review of the analysis outputs suggests that the evaluated models operated within acceptable safety boundaries in this educational context, with a complete absence of unethical language or inappropriate tone. This does not replace a formal safety audit, but provides preliminary evidence of alignment with the principles of trustworthiness and human-centered oversight outlined in the NIST AI Risk Management Framework and UNESCO’s guidance on generative AI in education^49,50^.

These results not only benchmark technical accuracy, but also underscore the potential of AI-assisted grading to transform assessment practices: making them more scalable, more consistent, and more responsive to student learning needs. However, achieving these benefits in practice requires careful policy design, instructor training, and ongoing human oversight to ensure fairness, transparency, and pedagogical alignment.

Although the empirical results presented in this study provide a snapshot of current model performance, an equally important contribution of this work is the proposal of a replicable evaluation framework for evaluating LLMs in higher education assessment contexts. As outlined in the methodology section, the proposed framework offers a transferable methodological structure that can be applied beyond the specific models evaluated here. By explicitly defining criteria for model selection, prompt formulation, evaluation measures, and cost estimation, the framework provides a generalizable blueprint that can be applied to future LLMs as they emerge. In this sense, the current comparison serves as an illustrative case study, while the framework itself remains relevant over time as a tool for researchers, educators, and institutions seeking to systematically assess AI-assisted grading in higher education.

## Conclusions

This study evaluated the effectiveness of recent LLMs for AI-assisted grading in a university-level course on data analytics and machine learning. By benchmarking six models on real and anonymized student deliverables, the analysis revealed that DeepSeek-R1 consistently provided the closest alignment with human evaluations, both in numerical grading and in the semantic quality of feedback. These results confirm the potential of advanced generative AI as a powerful complement to traditional assessment processes in technical higher education.

From a methodological perspective, this study contributes a replicable evaluation framework for assessing LLMs in academic contexts, combining statistical reliability tests for numeric grades, multi-level lexical and embedding-based similarity metrics for textual feedback, complementary qualitative analysis, and a standardized cost assessment. This methodology has been designed to serve as a reusable benchmark for evaluating the performance of future LLMs as they emerge and it can be adapted across disciplines, offering researchers and practitioners a structured approach to assess emerging AI models. At the same time, the results underscore current limitations: even the best-performing models occasionally missed subtle but pedagogically relevant errors, reinforcing the need for hybrid human–AI evaluation workflows, where quantitative similarity metrics combined with a qualitative post-hoc instructor reviews, allows institutions to separate “textual similarity” from pedagogical adequacy and to deploy LLM feedback as instructor-reviewed drafts in a “human-in-the-loop” workflow.

Beyond technical performance, the findings carry important pedagogical and institutional implications. AI-assisted grading can enhance formative assessment by providing more immediate and consistent feedback, allowing students to iterate on their learning more effectively. For instructors, these tools reduce repetitive grading workloads, enabling greater focus on mentoring and personalized guidance. At the institutional level, the study highlights trade-offs between performance and cost, suggesting that open-source or locally deployed models may offer a sustainable path for large-scale adoption, provided that compliance with GDPR and quality assurance standards is maintained.

Looking ahead, future research should advance in four directions. First, prompt engineering strategies should be systematically refined to optimize grading instructions and better align AI outputs with pedagogical objectives. Second, fine-tuning LLMs on domain-specific educational data could improve grading reliability and contextual accuracy, although challenges related to computational cost and accessibility remain. Third, integrating LLM-as-a-judge frameworks or meta-evaluation mechanisms may enhance assessment robustness by introducing a secondary evaluative layer within the multi-dimensional evaluation pipeline. Fourth, future work should extend the current framework by establishing more formalized and scalable approaches to feedback-quality measurement, reinforcing the qualitative dimension through systematic evaluation of specificity, actionability, and contextual depth. Further studies should also examine student’s and instructor’s perceptions of AI feedback, as trust, acceptance, and ethical concerns will ultimately influence its practical adoption.

In conclusion, AI-assisted grading should not be viewed as a replacement for human evaluation, but as a transformative addition to the educational technology landscape. Properly integrated into higher education, LLMs can support scalable, fair, and timely assessment practices, while human oversight ensures contextual nuance, fairness, and pedagogical depth. By pursuing continued research and responsible deployment, higher education institutions can leverage generative AI not only to increase efficiency but also to advance pedagogical innovation and institutional resilience in the digital era.

## Data Availability

Due to the sensitive nature of the primary practices and deliverables used in this research, individual-level data cannot be publicly shared. Please direct data access inquiries to the corresponding author, requests will be reviewed on a case-by-case basis to ensure compliance with ethical and privacy standards.

## Appendix A Representative annotated examples

In this section a selection of representative examples from all LLMs is presented:

- **Llama 3.3, P1, case 73.** Manual grade: 8. LLM grade: 82. Manual feedback: “Notebook is not running properly... data was not cleaned nor question addressed properly.” LLM feedback: “The code runs without major errors... The data loading and cleaning steps are well-performed.” Both adjudicators marked this as a clear case of missed concrete mistakes, flattened reasoning, large grading divergence, and informative but contextually shallow feedback.
- **DeepSeek-R1, P1, case 42.** Manual grade: 8. LLM grade: 50. The model explicitly noted that the 2024 data only extended to August and that the student failed to adjust the comparison period for fair year-over-year analysis. This makes it a strong example of a minor but important methodological mistake being identified only partially in the feedback but not weighted strongly enough in the final score.
- **gpt4oMini, P1, case 25.** Manual grade: 32. LLM grade: 79. The manual review described the submission as incomplete and wrong, with missing steps such as properly cleaning the data, missing answers to the main questions, and missing statistical tests. GPT-4o mini still framed the notebook as broadly workable, making this a clear example of an important weaknesses being underweighted in the final score.
- **gpt4o, P1 case 73** Manual grade: 28. LLM grade: 72. The manual reviewer stressed that the notebook was not running properly and that the required questions were not properly addressed, while GPT-4o still presented the work as a structured submission with room for improvement rather than a fundamentally unsuccessful one.
- **DeepSeek-V3, P1 case 61.** Manual grade: 36. LLM grade: 75. The manual review emphasized that the data had not been properly cleaned, that wrong distributional assumptions invalidated the use of a t-test, and that the resulting conclusions were biased. DeepSeek-V3 still produced a relatively positive rubric-based evaluation, illustrating how contextual and methodological problems could be recognized only weakly in the grade.
- **Llama 3.1, P1 case 45** is a useful example of apparent agreement masking a substantive difference in feedback content. Both the manual reviewer and the model assigned the same score (82/100), suggesting strong alignment at the grading level. However, the manual evaluation was more selective and context-sensitive, combining praise for the professional narrative and explanations with criticism of over-complicated analysis, insufficiently informative outputs, weak group-comparison statistics, and missing profit-margin analysis. By contrast, the LLM feedback remained more generic and rubric-shaped, emphasizing broadly appropriate data handling, exploratory analysis, and visualization quality without reflecting the same specific methodological reservations. This case therefore illustrates that similar grades do not necessarily imply similar evaluative reasoning.
- **Llama 3.3, P1 case 19.** Manual grade: 82. LLM grade: 82. BERTScore F1: 0.17. This case shows that semantic divergence can remain substantial even when grades are identical, and both adjudicators still marked it as missing subtle arguments and lacking contextual depth.
- **gpt4oMini, P2, case 9.** Manual grade: 28. LLM grade: 75. In this case, the LLM produced lengthy and well-structured feedback that appeared useful in terms of the rubric. However, the comments remained at the level of generic advice on data handling and modeling. The feedback included several suggestions related to preprocessing and model evaluation and correctly mentioned potential issues in data handling. Nevertheless, it failed to identify a methodological issue that also constituted a minor but important mistake highlighted in the manual evaluation: the RMSE comparison and resulting conclusions were invalid because the two models (linear regression and decision trees) were evaluated on differently aggregated data. Although the feedback appeared detailed and actionable, it did not address this analytical flaw, which ultimately determined the instructor’s grade: *’...The data is correctly loaded and merged, although variable naming could be clearer and the preprocessing stage would benefit from additional checks (e.g., explicit handling of missing values) and improved documentation. The choice of predictive models is appropriate and the reasoning behind the selection is explained, but the evaluation could be strengthened through more systematic parameter tuning (e.g., grid search or cross-validation) and the inclusion of additional performance metrics such as confusion matrices or classification reports. While the technical implementation is mostly sound, the business interpretation of the results remains underdeveloped, with limited discussion of how model performance translates into practical insights or decision-making implications. The report is generally well structured and the visualizations are useful, but some figures lack clear labels and the explanatory text could be expanded to make the analytical workflow easier to follow. Overall, the work shows good progress but would benefit from stronger documentation, more rigorous model evaluation, and deeper interpretation of the results.(...)’*

## Appendix B Prompts used as input

The prompts for Practice 1 and Practice 2 were collaboratively selected with the instructors and subsequently employed as default input queries in the automated evaluation of the models. They are included in this appendix to support reference and ensure reproducibility within the framework of this study.

### Prompt P1:

Evaluate the following Jupyter notebook for a Data Analytics practice for a University degree (be strict and brief).

Consider the following criteria:

1. Code correctness: Does the code run without errors? Is it efficient and well-structured?
2. Data handling: Are data loading, cleaning, and manipulation techniques appropriate and effective?
3. Analysis techniques: Are the chosen analysis methods suitable for the task? Are results interpreted correctly?
4. Visualization: Are the visualizations clear, informative, and well-labeled? Does the charts have a consistent clear color coding? Any chart without proper explanations or not a consistent color coding will penalize the final grade.
5. Explanation and documentation: Are the code and all analysis results well-explained and documented?
6. Are enough statistics and/or statistical tests to confirm the main claims?

Provide a short (10-20 lines) comprehensive evaluation with specific feedback and suggestions for improvement.

Finally include at the end an strict overall grade out of 100, in a new line and with this format: Grade: xx,

Penalize unclear variable names, missing comments, or lack of documentation.

Penalize the use of unsuitable methods or missing steps in the workflow.

Penalize weak or missing interpretations of results.

This is the student’s assignment deliverable:

*- The student deliverable is automatically inserted here -*

### Prompt P2:

Evaluate the following Jupyter notebook for a Data Analytics practice focused on machine learning, it is for a University degree (be strict and brief).

Consider the following criteria:

1. Data Handling & Preprocessing: Uses appropriate data, performs quality checks, and applies effective feature engineering.
2. Model Selection & Evaluation: Chooses suitable ML models, optimizes performance, and justifies evaluation metrics.
3. Business Insights & Decision-Making: Translates model results into actionable business recommendations.
4. Report Quality & Presentation: Communicates findings clearly with well-structured reports, visuals, and reproducible documentation.
5. Visualization: Are the visualizations clear, informative, and well-labeled? Does the charts have a consistent clear color coding? Any chart without proper explanations or not a consistent color coding will penalize the final grade.
6. Explanation and documentation: Are the code and all analysis results well-explained and documented?

Provide a short (10-20 lines) comprehensive evaluation with specific feedback and suggestions for improvement.

Penalize unclear variable names, missing comments, or lack of documentation.

Penalize the use of unsuitable methods or missing steps in the workflow.

Penalize if the training and test sets and sizes are not properly selected

Penalize weak or missing interpretations of results.

Finally include at the end an strict overall grade out of 100, in a new line and with this format: Grade: xx,

This is the student’s assignment deliverable:

*- The student deliverable is automatically inserted here - *

## Appendix C Sample of a LLM-generated evaluation report

This section contains a randomly selected example of an LLM assessment for Practice 1 produced by DeepSeek-R1:

“Okay, let’s start evaluating this submission. The first thing I notice is the structure. The student divided the work into steps like data collection, preprocessing, analysis for each question, and additional questions. That’s good for organization. But looking at the code, there are some issues. For example, they used ’pip install rich’ which isn’t standard in Jupyter notebooks and might cause errors if not handled properly. Also, some variable names like ’df_tot’ and ’nooutmem’ are unclear, making the code harder to follow.

Data handling seems okay. They merged datasets and handled outliers by adjusting sales and profits based on average values. However, the outlier detection process is a bit manual and lacks thorough explanation. For instance, when they adjusted the Sony Memory Card’s sales, they didn’t explain why they chose that method beyond a quick mention. Also, dropping rows with zero sales but high profit is correct, but the reasoning is brief.

Visualizations are a mixed bag. They created various plots like bar charts, boxplots, and violin plots, which is good. However, many plots lack proper labels or consistent color coding. For example, some bar charts don’t specify units on axes, and the use of ’skyblue’ and ’lightgreen’ might not be consistent across all figures. The heatmaps for correlation are a good touch, but they could benefit from better annotations or explanations.

Analysis techniques: The student used Mann-Whitney U tests and *t*-tests, which are appropriate for non-parametric data. However, in Q2, they used a *t*-test for comparing profits between categories, which might not be suitable if the data isn’t normally distributed. They should have stuck with non-parametric tests throughout. Also, the interpretation of statistical results is sometimes lacking depth. For example, they mention p-values but don’t discuss effect sizes or confidence intervals.

Documentation and explanations are present but could be improved. The code has some comments, but many steps are under-explained. For instance, the handling of the marginal profit outlier at index 3963 involves a proportion calculation that’s not well-documented. The narrative in the markdown cells helps, but some sections are verbose without adding clarity, like the EDA parts which repeat what’s evident from the plots.

Statistical rigor: They performed tests for Q1 and Q3 but in Q2, the analysis of the e-marketing campaign’s impact is weak. The student compared before and after July but didn’t account for seasonal trends adequately. The Mann-Whitney test here might not be the best choice without considering other factors. Also, the conclusion for Q2 correctly states no significant boost for Technology but lacks depth in explaining why other categories also increased.

Additional questions: The answers are correct but very brief. For example, calculating total revenue for 2024 is straightforward, but more context or explanation of the steps would be better. The use of ’rich’ and ’tabulate’ for pretty printing is nice but not necessary and could complicate reproducibility if dependencies are missing.

Overall, the student demonstrates a good grasp of data analysis steps but needs to improve code clarity, visualization consistency, and statistical method justification. The conclusions are supported, but some parts need deeper analysis and better documentation.

**Evaluation:**

1. **Code Correctness (7/10):**

- Runs but has readability issues (e.g., unclear variable names like nooutmemm, df_tot).
- Some redundant operations (e.g., repeated filtering for outlier handling).
- Missing comments for complex operations (e.g., profit adjustment logic).

2. **Data Handling (8/10):**

- Effective outlier detection and correction (e.g., Sony Memory Card).
- Proper merging and date parsing.
- Missing deeper validation of cleaned data distributions.

3. **Analysis Techniques (7/10):**

- Appropriate use of Mann-Whitney U tests for non-parametric data.
- **Major Issue:** Used *t*-tests for Q3 (profit comparisons) without checking normality, violating test assumptions.
- Limited exploration of seasonality in Q2 (e-marketing impact).

4. **Visualization (6/10):**

- Multiple plots but inconsistent labeling (e.g., missing units, unclear axes in time series).
- Color coding is applied but not always consistent across figures.
- Some plots lack clear interpretation (e.g., violin plots without explanation).

5. **Explanation (6/10):**

- Adequate narrative for main steps but lacks depth in critical areas (e.g., marginal profit adjustments).
- Statistical results reported without confidence intervals or effect sizes.
- Conclusions are data-driven but occasionally oversimplified (e.g., Q2 campaign impact).

6. **Statistical Rigor (7/10):**

- Tests address main hypotheses but lack robustness checks (e.g., normality/heteroscedasticity).
- No multiple-testing correction applied.
- Q2 analysis lacks counterfactual (e.g., comparing 2023 trends to previous years).

7. **Improvements Needed:**

- Use consistent variable names and add comments for complex logic.
- Replace *t*-tests with non-parametric alternatives (e.g., Kruskal-Wallis).
- Improve plot annotations and adopt a cohesive color scheme.
- Discuss effect sizes and limitations of statistical tests.

**Grade: 70**

**Penalties:** -10 (statistical method flaws), -8 (visualization/documentation), -7 (code quality), -5 (partial result interpretation).”

## Appendix D Wilcoxon signed-rank test results

To rigorously assess the performance of the Large Language Models against the human benchmark, we conducted a Wilcoxon Signed-Rank Test on the paired data (LLM grade vs. Manual grade) for each of the six models. The results, presented in Table 9, indicate the degree of statistically significant difference between the median grade assigned by each LLM and the median grade assigned by the human rater, thus quantifying the level of agreement between AI-assisted and manual grading.

**Table 9:** Wilcoxon Signed-Rank test comparing LLMs grades with manual grades.

Practice	Model	p-value	Significant (99%)
P1	gpt4oMini	0.0000	Yes
P1	gpt4o	0.0000	Yes
P1	Llama-3.1	0.0000	Yes
P1	Llama-3.3	0.0000	Yes
P1	DeepSeek-R1	0.2904	**No**
P1	DeepSeek-V3	0.0000	Yes
P2	gpt4oMini	0.2391	**No**
P2	gpt4o	0.3741	**No**
P2	Llama-3.1	0.0001	Yes
P2	Llama-3.3	0.0001	Yes
P2	DeepSeek-R1	0.8008	**No**
P2	DeepSeek-V3	0.3864	**No**

## Footnotes

1 General Data Protection Regulation (GDPR) is a unified European law for the processing of personal data of individuals within the European Union. Regulation (EU) 2016/679 of the European Parliament and of the Council of 27 April 2016 on the protection of natural persons with regard to the processing of personal data and on the free movement of such data. https://eur-lex.europa.eu/eli/reg/2016/679/oj/eng.

2 In the context of LLMs, a token is the basic unit of text that the model processes. Different LLMs use different tokenization algorithms, While many tokens are single common words, like “the”, “cat” or “car”, many other are parts of words, individual characters, punctuation marks, or even spaces.

3 LLMs have limited input context windows that typically are 32 K, 64 K or 128 K tokens. The latest LLMs versions include larger context windows that have up to 1M tokens.

4 Microsoft OpenAI gpt-4o-2024-08-06, https://platform.openai.com/docs/models/gpt-4o.

5 Microsoft OpenAI gpt-4o-mini-2024-07-18, https://platform.openai.com/docs/models/gpt-4o-mini.

6 Meta AI Llama 3.1-70B, https://www.llama.com/docs/model-cards-and-prompt-formats/llama3_1/.

7 Meta AI Llama 3.3-70B, https://www.llama.com/docs/model-cards-and-prompt-formats/llama3_3/.

8 DeepSeek-R1, https://github.com/deepseek-ai/DeepSeek-R1.

9 DeepSeek-V3, https://github.com/deepseek-ai/DeepSeek-V3.

10 Word embeddings were obtained from the pre-trained model *word2vec-google-news-300*, this model provides 300-dimensional vectors for a vocabulary of approximately 3 million words and phrases, see^54^.

11 We have used $en\_core\_web\_md$ model. This is spaCy’s medium-sized English language pipeline, trained on a mix of web-written text (blogs, news, comments) with a balance between model size and accuracy, available from https://spacy.io/models/en#en_core_web_md.

12 The Stanford Question Answering Dataset (SQuAD) is a premier machine reading comprehension benchmark featuring 100,000+ human-posed questions on Wikipedia articles comprises over 500 articles with 100,000+ question-answer pairs, widely used to train and evaluate NLP models.

13 The empirical thresholds in this dataset were: Q3 of absolute grade difference: 30.0, Q1 of manual-vs-LLM BERTScore F1: 0.221

14 In post-hoc reported results, both practices are aggregated and positive cases are counted whenever at least one instructor identified the issue.
